# Application of experimentally verified transcription factor binding sites models for computational analysis of ChIP-Seq data

**DOI:** 10.1186/1471-2164-15-80

**Published:** 2014-01-29

**Authors:** Victor G Levitsky, Ivan V Kulakovskiy, Nikita I Ershov, Dmitry Yu Oshchepkov, Vsevolod J Makeev, T C Hodgman, Tatyana I Merkulova

**Affiliations:** Institute of Cytology and Genetics of the Siberian Division of Russian Academy of Sciences, Lavrentieva Prospect 10, Novosibirsk, 630090 Russia; Novosibirsk State University, Pirogova 2, Novosibirsk, 630090 Russia; Engelhardt Institute of Molecular Biology, Russian Academy of Sciences, Vavilova str. 32, Moscow, 119991 Russia; Department of Computational Systems Biology, Vavilov Institute of General Genetics, Russian Academy of Sciences, Gubkina str. 3, Moscow, 119991 Russia; Multidisciplinary Centre for Integrative Biology, School of Biosciences, University of Nottingham, Sutton Bonington, LE12 5RD UK

**Keywords:** ChIP-Seq, EMSA, Transcription factor binding sites, FoxA, SiteGA, PWM, Transcription factor binding model, Dinucleotide frequencies

## Abstract

**Background:**

ChIP-Seq is widely used to detect genomic segments bound by transcription factors (TF), either directly at DNA binding sites (BSs) or indirectly via other proteins. Currently, there are many software tools implementing different approaches to identify TFBSs within ChIP-Seq peaks. However, their use for the interpretation of ChIP-Seq data is usually complicated by the absence of direct experimental verification, making it difficult both to set a threshold to avoid recognition of too many false-positive BSs, and to compare the actual performance of different models.

**Results:**

Using ChIP-Seq data for FoxA2 binding loci in mouse adult liver and human HepG2 cells we compared FoxA binding-site predictions for four computational models of two fundamental classes: pattern matching based on existing training set of experimentally confirmed TFBSs (oPWM and SiteGA) and *de novo* motif discovery (ChIPMunk and diChIPMunk). To properly select prediction thresholds for the models, we experimentally evaluated affinity of 64 predicted FoxA BSs using EMSA that allows safely distinguishing sequences able to bind TF. As a result we identified thousands of reliable FoxA BSs within ChIP-Seq loci from mouse liver and human HepG2 cells. It was found that the performance of conventional position weight matrix (PWM) models was inferior with the highest false positive rate. On the contrary, the best recognition efficiency was achieved by the combination of SiteGA & diChIPMunk/ChIPMunk models, properly identifying FoxA BSs in up to 90% of loci for both mouse and human ChIP-Seq datasets.

**Conclusions:**

The experimental study of TF binding to oligonucleotides corresponding to predicted sites increases the reliability of computational methods for TFBS-recognition in ChIP-Seq data analysis. Regarding ChIP-Seq data interpretation, basic PWMs have inferior TFBS recognition quality compared to the more sophisticated SiteGA and *de novo* motif discovery methods. A combination of models from different principles allowed identification of proper TFBSs.

**Electronic supplementary material:**

The online version of this article (doi:10.1186/1471-2164-15-80) contains supplementary material, which is available to authorized users.

## Background

Identification of transcription regulatory elements in a genome is an actively evolving topic in modern molecular biology. The major class of these elements is represented by transcription factor (TF) binding sites (TFBSs), short DNA segments of 10-20 bp specifically recognized by TFs. Modern high-throughput techniques, such as chromatin immunoprecipitation (ChIP) followed by microarray hybridization (ChIP-chip) or by massively parallel sequencing (ChIP-Seq), allow genome-scale mapping of TF occupancy in a given cell type and state [1]. To date, thousands of binding loci for a large number of TFs have been revealed for various cell types [2]. However, both ChIP-Seq and ChIP-chip technologies are not able to distinguish direct TF binding to DNA from indirect binding mediated by other chromatin proteins including other TFs bound to cognate DNA sites (the so-called tethered or “piggy back” binding) [1, 3]. Prediction of the genome-wide TF binding landscape, i.e. identification of the entire set of TFBSs existing in a particular genome irrespective to the cell type and state, is also unlikely to be done without proper TFBS modeling *in silico*. Furthermore, ChIP-Seq identifies exact locations of TFBSs only indirectly and cannot discriminate between closely spaced multiple sites within DNA segments of hundreds of base pairs [4].

To identify TFBSs in a given sequence one applies computational methods for their recognition. A myriad of such methods exists today, falling into two main classes [5, 6]. The first class is based on pattern matching, also called motif finding. In this case, the TFBS recognition model is constructed on an independent training set of TFBS sequences obtained from conventional gene-by-gene experimental studies. The pattern is often represented as a positional weight matrix (PWM) which assumes that nucleotides in BS sequence additively and independently contribute to the total binding energy [5]. The PWM is widely used for TFBSs recognition in genomic sequences, e.g. to interpret ChIP-Seq data. A number of information resources contain the ready-to-use TFBS matrices, namely, TRANSFAC [7], JASPAR [8], ARTSITE [9], HOCOMOCO [10] etc. The performance of conventional PWMs can be improved if dependencies between adjacent positions are taken into account, e.g. using so-called dinucleotide PWMs [11]. The next improvement of this approach is a proper selection of matrix length, i.e. construction of optimized PWM (oPWM) [12, 13]. More sophisticated and much less commonly used methods do not include “the additivity assumption”, i.e. nucleotides in different positions may depend on each other [14, 15]. Among these methods is our previously developed SiteGA, well-proven in recognition of various TFBSs [12].

The second class of methods is oriented towards *de novo* pattern detection, and referred to as motif discovery, also often utilizing PWMs as the TFBS model. Initially, motif discovery was proposed to identify TFBSs in promoter sequences of co-regulated or orthologous genes. Although motif discovery algorithms have been shown to work successfully in bacteria and yeast, they performed significantly worse in higher organisms [16]. However, the motif discovery approach has become of extremely high value with the emergence of ChIP-chip/ChIP-Seq technologies [17, 18]. Currently, many variations of such methods exist, some of them are presented in well-known resources. ChIPMunk [19] and diChIPMunk [20] belong to this class. Using the basic PWM model ChIPMunk performed nicely in several independent benchmarks [21, 22], including the recent one of the DREAM consortium [23]. diChIPMunk uses the same engine as ChIPMunk to produce dinucleotide PWMs.

It is of great interest to compare the performance of the motif discovery and motif finding approaches applied to the same experimental data. However, no such studies have been carried out until now. Moreover, a comparative analysis of the advantages and shortcomings of different methods is hampered by the lack of direct experimental verification of predicted TFBSs.

Using a FoxA2 ChIP-Seq data for mouse adult liver chromatin [24] and human hepatoma cell line chromatin [25] we conducted a comparative assessment of oPWM and SiteGA (pattern-matching models), ChIPMunk, and diChIPMunk (pattern-detection models), which was accompanied by experimental verification.

FoxA2 is a member of the FoxA subfamily of winged helix/forkhead box (Fox) transcription factors playing important roles at different stages of mammalian life cycle, including early development, organogenesis, and metabolism and homeostasis in the adult [26]. FoxA2 was shown to be a pioneer transcription factor [27], thus indirect (mediated by other DNA-binding proteins) binding of FoxA2 to chromatin should not be a major event. With the independent human and mouse liver ChIP-Seq datasets available FoxA2 is one of the most convenient TFs to compare different computer approaches for prediction of TFBSs.

## Results

### Identification of FoxA binding sites in promoter ChIP-Seq loci

Initially, to compare the performance of pattern matching and pattern detection approaches for TFBS prediction in the context of ChIP-Seq data, we applied oPWM and SiteGA (as representatives of the former class) as well as ChIPMunk and diChIPMunk (as representatives of the latter class) to analyze a dataset of 4455 FoxA2-binding loci (ChIP-Seq peaks with read coverage of at least 15) in mouse adult liver chromatin [24].

**Figure 1:**
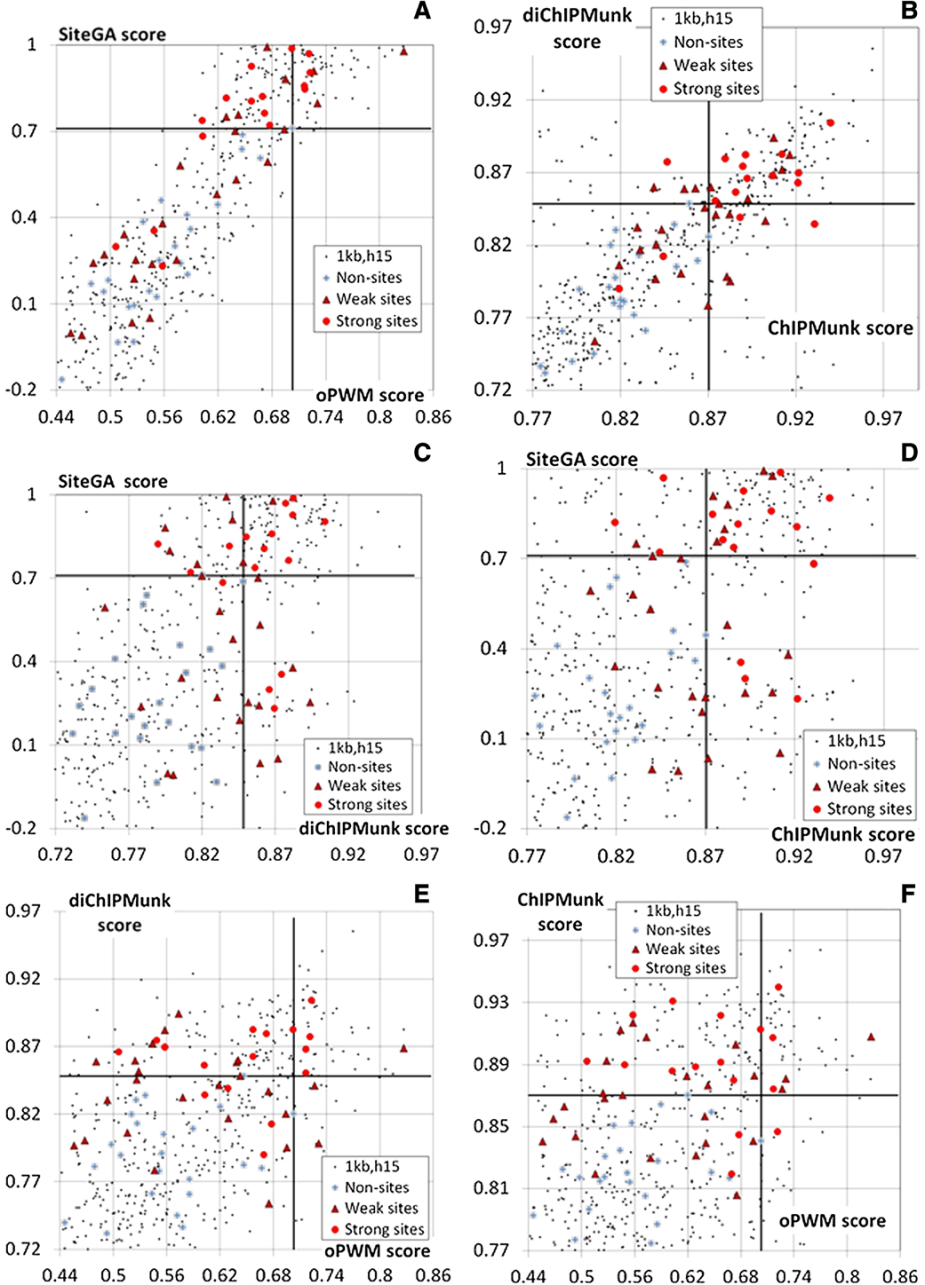
**Scores of different FoxA BS recognition models for TFBSs derived from ChIP-Seq data [**[24]**].** Six panels from **A** to **F** denote all possible pairwise combination of ChIPMunk, diChIPMunk, SiteGA and oPWM models. Black dots denote the sample of 466 potential BSs: **(A)** sites mapped in promoter regions located in 1000 bp upstream regions of RefSeq genes; **(B)** binding sites lying in peak regions with peak height of 15 or higher. The 64 BSs selected for experimental verification are shown as grey crossed squares, brown triangles and red circles, corresponding to the EMSA scores below 0.25 (non-sites), in the range from 0.25 to 0.75 (weak sites) and above 0.75 (strong sites). Solid lines mark the model thresholds selected to discard non-sites.

To produce a subset of data for experimental verification we restricted the search to FoxA2-binding loci that overlapped with 1 kb upstream regions of RefSeq genes (mm8 assembly) and had coverage at least 15 (301 promoters). Totally 466 putative FoxA BSs were predicted in these regions. Each BS was characterized by a set of four scores corresponding to the four models used. The thresholds applied were very low, so that among selected putative BSs were those with non-consistent functionality. The pairwise comparison of scores (Figure 1) showed a good agreement between models of the same class (pattern match or pattern detection). Thus, there was a strong correlation between predictions of oPWM/SiteGA (Figure 1A, Pearson correlation coefficient 0.872) and ChIPMunk/diChIPMunk (Figure 1B, 0.708). The agreement between other pairs of models was notably lower (with the highest correlation coefficient of 0.625 for SiteGA/diChIPMunk; Figure 1C). Although a considerable portion of points still landed close to the scatterplot diagonal, i.e. many sites are similarly scored even by principally differing models (Figure 1C-F), there was a considerable number of sequences with a significantly higher score assigned by only one of the models, i.e. displaying incompatible predictions of different models. Thus, a special interest was to determine whether these sites were able to bind TFs in practice.

### Experimental verification of predicted FoxA binding sites by EMSA

**Figure 2:**
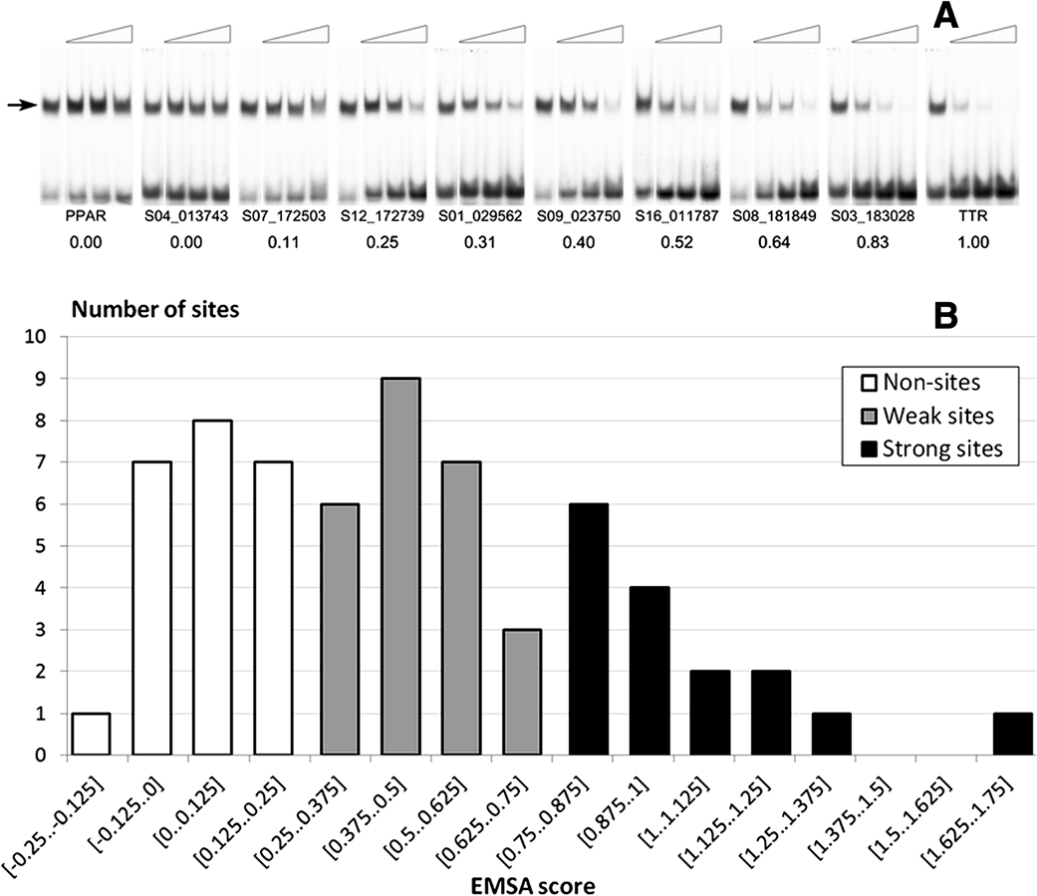
**Experimental verification of putative FoxA sites by EMSA. A** – EMSA competition of oligonucleotides containing predicted FoxA sites, with labeled TTR probe for binding to recombinant GST-FKH-FoxA2 (demonstrative autoradiographs). The ascending concentrations (2, 5, and 20 ng) of cold competitors are shown as triangles at the top of the figure, their IDs and resulting relative EMSA scores are shown at the bottom. The band corresponding to the DNA-protein complex is marked by the arrow. Unlabeled TTR and PPAR oligonucleotides were used as positive and negative controls of protein binding. **B** – Distribution of EMSA scores for 64 potential FoxA binding sites selected for EMSA verification. Selected BSs had peak height of at least 15 and were located in 1000 bp upstream regions of RefSeq genes. The X and Y axes denote the EMSA score and the number of sites predicted at a specific EMSA-score threshold. EMSA scores are rough estimates of TFBS affinities relative to that of the positive control site (referred to as the EMSA scores listed in Additional file 1: Table S1). White, grey and black columns denote the EMSA scores below 0.25 (non-sites), 0.25 to 0.75 (weak sites) and above 0.75 (strong sites).

Out of 466 BSs predicted in promoter regions, 64 were arbitrarily selected for experimental verification by EMSA. Among them there were the sites quite differently evaluated by the models used (Figure 1). The main advantage of EMSA is unambiguity in interpretation of the results. This method records the fact of the TF binding to the oligonucleotide corresponding to a predicted site, thereby making it possible to set the threshold directly and restrict the false-positives [3]. In this study, double-stranded oligonucleotides for 64 selected FoxA sites were individually added in ascending concentrations as cold competitors to the binding reaction of labeled oligonucleotide corresponding to a well-known FoxA site from mouse *Ttr* promoter [28] with purified GST-FKH-FoxA2 protein. A representative autoradiograph of separated complexes is shown on Figure 2A.

Rough estimate of TFBS affinities relative to that of TTR oligonucleotide were calculated (further referred to as the EMSA scores listed in Additional file 1: Table S1). It was found, that the distribution of estimated the EMSA scores for 64 tested sites was essentially continuous (Figure 2B; Additional file 2: Figure S1), which agrees with the previous data for other TFs [29, 30]. Based on the experimental results, all the studied sequences were divided into three groups: a) non-sites, EMSA scores below 0.25; b) weak sites, EMSA scores from 0.25 to 0.75 and c) strong sites, EMSA scores above 0.75 (Figure 1). Thus, the group of non-sites consisted of sequences whose ability to bind to GST-FKH-FoxA2 was not significantly different from the unrelated sequence.

Comparison of the predictions and the experimental data are shown in Figure 1. Most of the non-sites receive low scores from all the models (the bottom left corner in the scatterplots, Figure 1). In the case of the strong and the weak sites the picture is different. As expected, models of the same class (pairs oPWM/SiteGA and ChIPMunk/diChIPMunk) predicted sites concentrating in the top right corner of the scatterplot (Figure 1A,B), i.e. the predictions were mostly consistent. At the same time, predictions were less consistent for motif discovery versus motif-finding approaches. There were many sites with high scores from one model and low scores from another (Figure 1C-F), e.g. SiteGA versus ChIPMunk predictions (Figure 1D). These models utilized different training sets, and algorithms (SiteGA accounts for mutual dependencies between arbitrary positions, while ChIPMunk does not). So, a combination of such fundamentally different models can be the most effective in analyzing genome-wide ChIP-Seq data.

For further analysis we selected EMSA score cutoff of 0.25, which allowed determination of the thresholds for prediction models on a common basis (Figure 2). These are 0.870, 0.848, 0.710 and 0.703 for ChIPMunk, diChIPMunk, SiteGA and oPWM, respectively. For each model, we selected the threshold value as the highest score for the subset of non-sites (Figure 1).

### Identification of FoxA binding sites in genome-wide ChIP-Seq data

**Figure 3:**
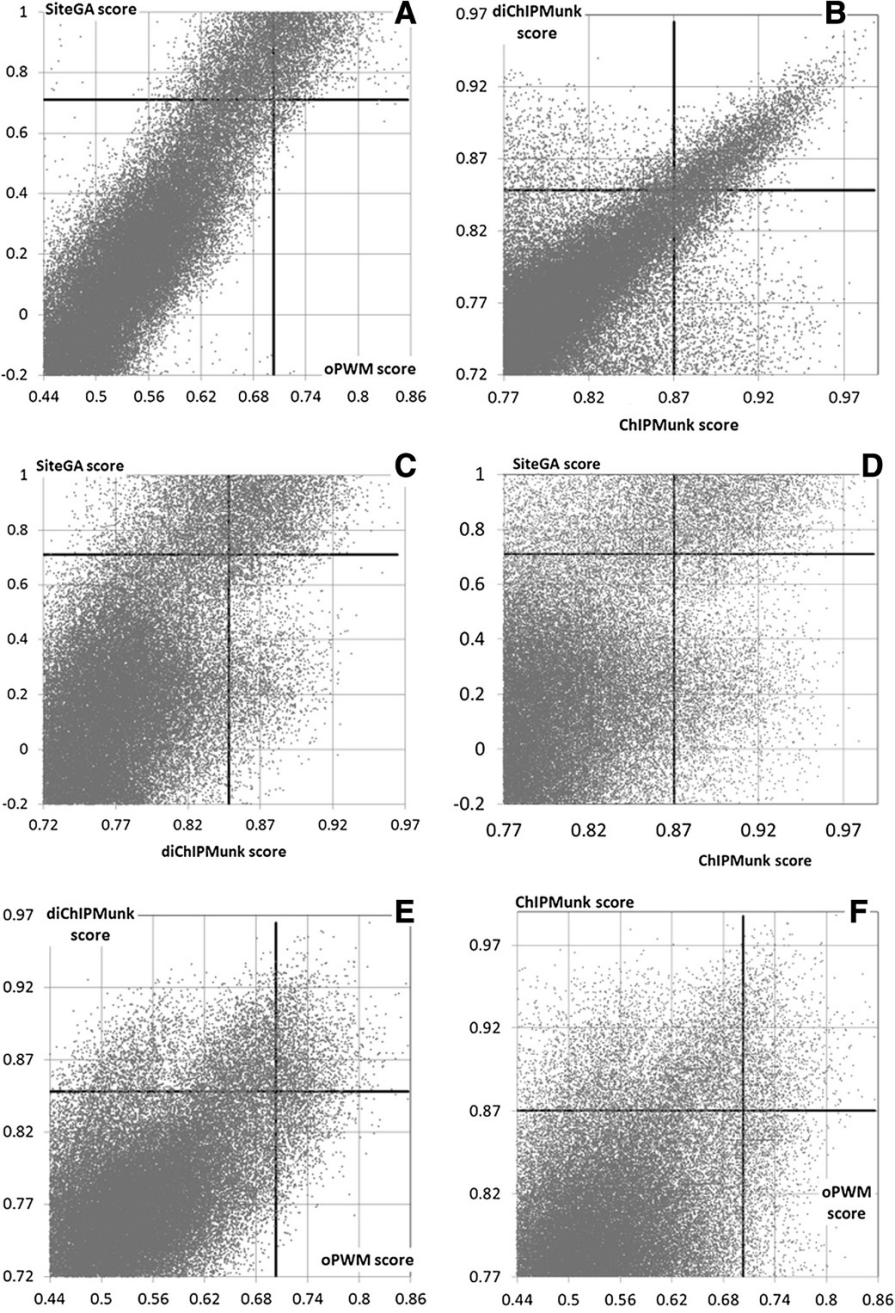
**The scatterplots of scores of different FoxA BS recognition models for BSs derived from ChIP-Seq data [**[24]**].** Six panels from **A** to **F** denote all possible pairwise combinations of ChIPMunk, diChIPMunk, SiteGA and oPWM models. Dots denote the subset of 49722 potential BSs that were mapped in ChIP-Seq peaks with heights of 15 and higher. Solid lines mark thresholds corresponding to the EMSA scores below and above 0.25.

Figure 3 shows scatterplots of scores of potential FoxA sites predicted in all 4455 FoxA2-binding loci [24]. As for the promoter regions, closely related models evaluated most of the sites in a similar manner (Figure 3A,B), whereas application of strongly differing approaches resulted in a substantial fraction of sites scoring highly by only one of them (Figure 3C-F). The use of thresholds, determined by EMSA (Figure 3, black lines), allowed us to discard sequences without threshold-passing predictions, i.e. possibly not capable to bind FoxA directly. As a result, SiteGA identifies 6884 reliable FoxA sites in 76.7% of peaks, ChIPMunk – 7000 in 82.7% of peaks, and diChIPMunk– 6079 in 78.7% of peaks. However, only 3008 FoxA sites in 45.1% of peaks are identified with oPWM approach, i.e. oPWM showed the weakest performance of all four models. It is worth noting that combined use of the models significantly increases the resulting number of the peaks with the identified sites. As expected, among all combinations the best was SiteGA/ChIPMunk with 90.0% of peaks (10040 sites) with BSs recognized by at least one model out of the pair. Another good combination was diChIPMunk/SiteGA with 88.9% peaks (8985 sites). A detailed analysis of SiteGA/ChIPMunk and diChIPMunk/SiteGA results showed that the majority of ChIP-Seq peaks contained more than one site (62.3% and 58.3%, respectively). These results were in agreement with existing observations that FoxA prefers to bind to clusters of sites in the regulatory regions of well-studied target genes [31–35].

The results show the high efficiency of the SiteGA model, as well as both participants from pattern-detection class (ChIPMunk and diChIPMunk). However, since the latter models were trained on the same data as used for the performance evaluation, the correct comparative assessment requires an additional independent control ChIP-Seq dataset.

### Application of TFBS models to the control FoxA ChIP-Seq dataset

The dataset described in [25] was taken as an independent control and contained 4367 FoxA2-binding loci, with read coverage of at least 10. In this dataset SiteGA, ChIPMunk, diChIPMunk and oPWM models recognized 5781 sites in 77.6% of peaks, 5629 sites in 81.5% of peaks, 4892 sites in 76.6% of peaks, 2394 sites in 43.0% of peaks respectively, showing almost the same performance as on Wederell’s data [24]. Note that oPWM again had the worst prediction rate.

**Figure 4:**
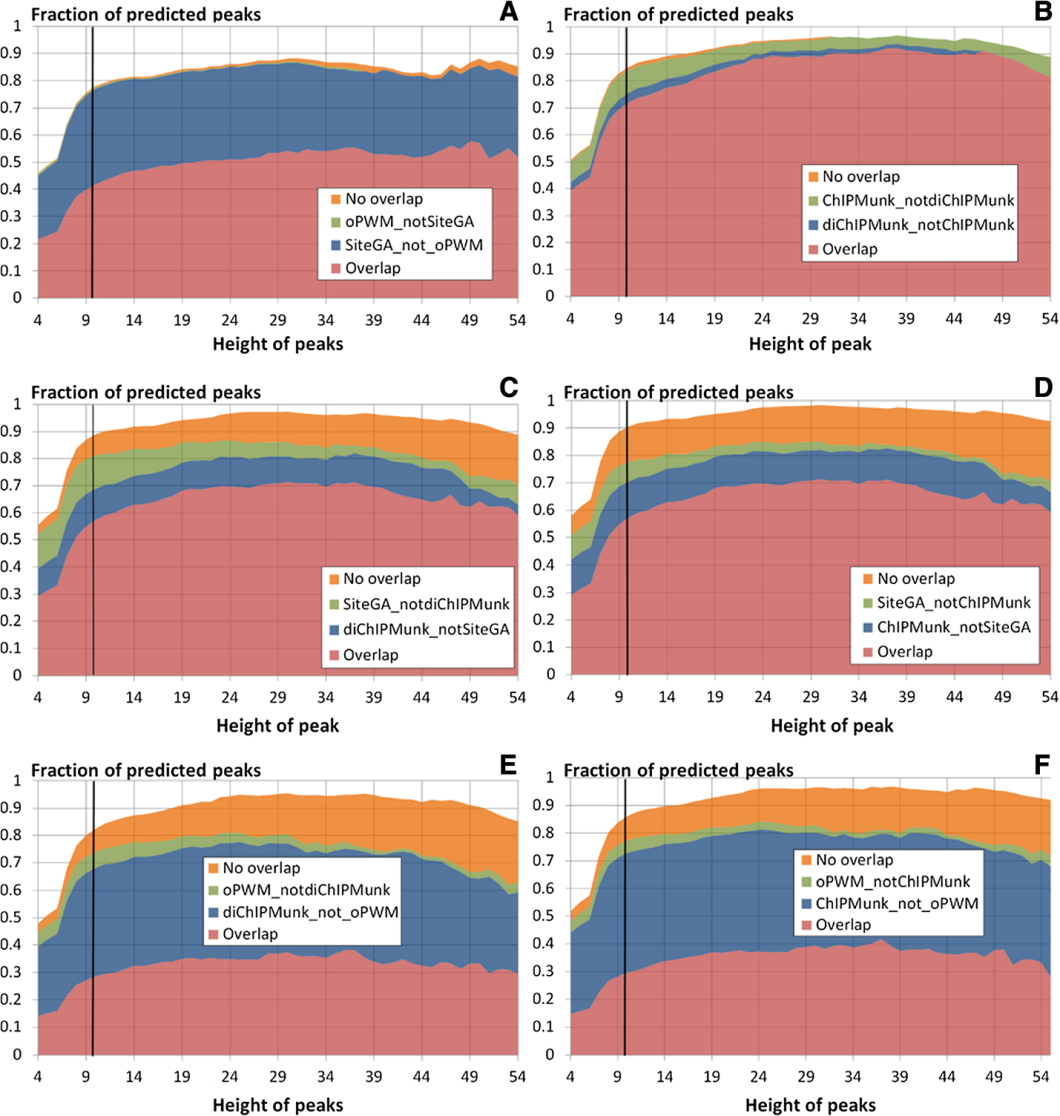
**The fraction of ChIP-Seq peaks** **[**[25]**] with recognized sites as a function of the peak height cut-off value.** Six panels from **A** to **F** denote predictions computed for all possible pairwise combinations of ChIPMunk, diChIPMunk, SiteGA and oPWM models. The X and Y axes show the fraction of peaks having at least one FoxA binding site recognized by the respective model and the peak height cut-off value. The panels from **A** to **F** show the comparisons between SiteGA and oPWM, ChIPMunk and diChIPMunk, SiteGA and ChIPMunk, SiteGA and diChIPMunk models, respectively. The following grouping of recognized TFBSs is used: loci with TFBSs recognized by both the first and second models (e.g. **D** – SiteGA & ChIPMunk, respectively) that either overlap or not, are marked by RED and ORANGE, respectively; loci with at least one FoxA binding site recognized by the first but not by the second model, or vice versa, are marked by GREEN and BLUE, respectively. Black vertical lines denote the coverage threshold (peak height of 10).

We expected the combination of models from different classes (pattern matching and pattern detection) would be more effective for analysis of genome-wide ChIP-Seq data. To estimate performance of pairwise combinations of our four models we computed the number of peaks with BSs recognized by each pairs of models. The following cases were separately processed: (a) peaks with at least one (overlapping) BS predicted by two models, (b,c) peaks with sites recognized by only one of the two models and (d) peaks with only non-overlapping site predictions of by two models (Figure 4). Note, that the total sum of these fractions reflects the total number of peaks where at least one site was found by any of the two tested models.

For all pairwise combinations the dependence of the fraction of recognized peaks from the peak height cut-off demonstrated a non-monotonic behavior, with significant growth from approximately 50% of recognized peaks at a cutoff value of 4 and a nearly uniform distribution at cutoff values of 10 and higher. Basically, this means that more erroneous peaks or weak sites are detected near the lower peak height of 4, supporting the choice of the peak height cutoff value of 10 for our study.

The models from the same class (two pattern matching or two pattern detection models) poorly complemented each other, giving a moderate rise in the number of peaks with recognized BSs for the ChIPMunk/diChIPMunk pair (joint recognition 85.0% vs. 81.5/76.6% for separate models, respectively; Figure 4B) and almost no effect for oPWM/SiteGA as SiteGA managed to recognize almost every peak detected by oPWM (joint recognition 78.2% vs. 77.6/43.0% for separate models, respectively; Figure 4A).

On the contrary, the combination of pattern-matching and pattern-detection models showed a substantial increase in the number of peaks with recognized BSs. Among all combinations the best again were SiteGA/ChIPMunk and SiteGA/diChIPMunk, with 90.7% and 89.1% peaks recognized by at least one model out of a pair, respectively (Figure 4C,D). More than a half of detected sites were recognized by two models simultaneously: 58.1% of sites (4679) and 58.9% (4196) for SiteGA/ChIPMunk and diChIPMunk/SiteGA, respectively (Figure 4C,D, red). The fractions of peaks solely detected by a particular model were 9.1% for SiteGA and 13.1% for ChIPMunk (Figure 4C, blue and green). For another combination, the respective fractions were 11.5% for SiteGA and 12.5% for diChIPMunk (Figure 4D, blue and green). The fractions of simultaneously detected peaks with non-overlapped sites were 8.9% and 8.4% (see Figure 4C,D, orange). A more detailed analysis of SiteGA/ChIPMunk and diChIPMunk/SiteGA results again shows that the majority of peaks contained more than one site, 54.0% and 50.1% respectively. These estimates are very similar to those computed above for Wederell’s ChIP-Seq data [24].

To get an estimate for the fraction of simultaneously detected peaks with non-overlapping sites expected by chance we performed a tenfold simulation on a random 1st order Markov chain background set that was generated by shuffling full-length peaks. We count observed and expected fractions of peaks with non-overlapped sites among all peaks detected by two models. For the SiteGA/ChIPMunk pair the expected fraction was 14.1% and observed one was 23.0% (p < 5E-12 according to the χ^2^ test for a 2×2 contingency table). This provides evidence that these non-overlapping predictions are likely nonrandom and are due to heterogeneity of the FoxA BSs sequences and their preference to form homotypic TFBS clusters.

Thus, usage of a pair of different models allowed both an increase in the number of peaks with precisely positioned sites, and identification of multiple sites within a single peak. The latter is quite important, since homotypic clusters of TFBSs are often found in the gene regulatory regions, underlying a number of mechanistic advantages [36, 37]. These may include favoring high-affinity cooperative TF binding and lateral diffusion of TF binding along regulatory regions, or simply increasing of the local TF concentration in the vicinity of these regions and thereby increase the probability of functional interactions [38–40].

Note that oPWM can also provide a benefit when SiteGA is not available. For example, oPWM slightly increased the number of peaks with detected BSs (4.6% only oPWM, 43.1% only ChIPMunk, and 5.9% only oPWM, 39.5% only diChIPMunk, Figure 4E,F: blue and green) and the number of peaks with non-overlapping sites detected by different models (8.6% and 8.3%, Figure 4E,F, orange).

### Comparison of constructed and existing FoxA TFBS models

**Figure 5:**
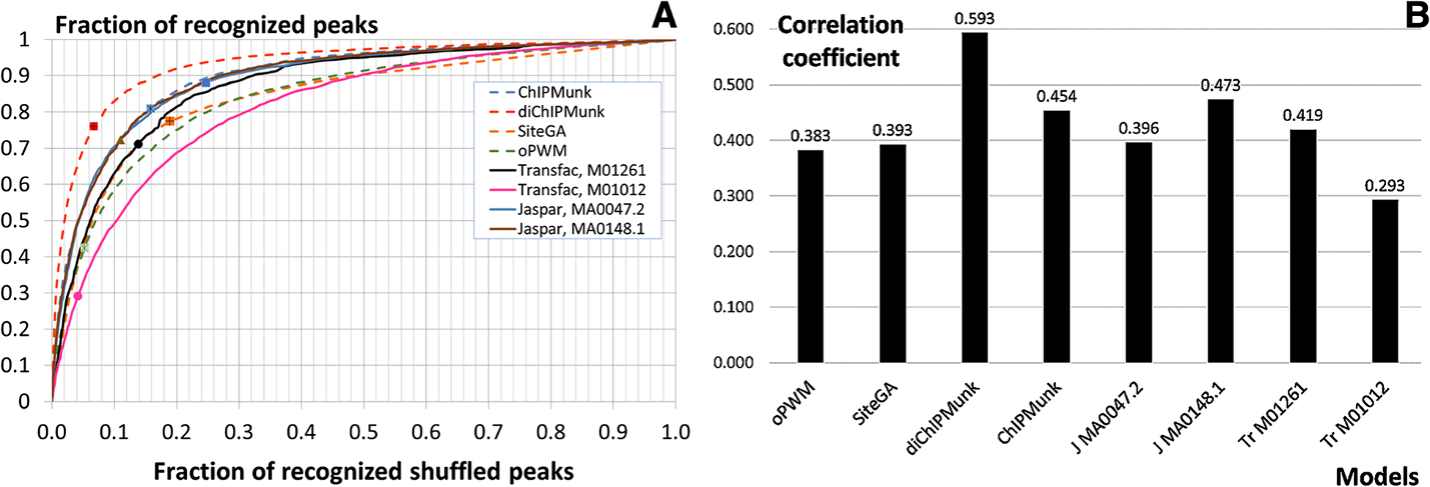
**ROC (Receiver operating characteristic) of TFBS models (A) and CC (correlation coefficient) (B).** **A** – ROC curves for 8 recognition models applied to ChIP-Seq data [25]. ‘J’ - JASPAR PWM, ‘Tr’ - TRANSFAC PWM. The fraction of recognized peaks (X axis) shows the true positive (TP) rate. The fraction of recognized shuffled peaks (Y axis) shows the false positive (FP) rate. Markers for each model show the point corresponding to the EMSA-derived threshold. **B** – the correlation coefficients reflect balance between TP and FP rates computed for thresholds derived from EMSA verification. The values of TP and TP rates are shown in ROC curves **(A)** by markers.

To compare the performance of the four models constructed in this study with known TFBS models we used four additional PWMs: MA0047.2 [41] and MA0148.1 [42] from JASPAR [8], M01261 [43] and M01012 [44] from TRANSFAC [7]. Among them only the latter was not derived from ChIP-Seq data. Receiver operating characteristic (ROC) curves were used to compare eight models with Wallerman dataset [25] (Figure 5A). False positive rates were estimated over the first order Markov chain background-set that was generated by shuffling full-length peak sequences. ROC curves close to the top left corner correspond to the models with better performance. Figure 5A clearly shows that the diChIPMunk model outperformed all others ones. This seems to be a consequence of the dinucleotide in contrast to mononucleotide statistics used by other ChIP-Seq-derived models. The TRANSFAC PWM M01012 showed the worst performance. To compare other models in detail, we computed the correlation coefficients (CC) that reflect the relationship between true and false positive rates [45]. The CC values were computed for the EMSA-based thresholds chosen earlier. EMSA thresholds for additional models were selected as described above (see Results section “Experimental verification of predicted FoxA binding sites by EMSA”). The ChIPMunk, JASPAR and M0148.1 matrices showed similar performance, leaving TRANSFAC M01261 and JASPAR MA0047.2 the worst among ChIP-Seq-derived models. Note that the performance of SiteGA was on a par with those for ChIP-Seq-derived models, outperforming other models not derived from ChIP-Seq data, oPWM and TRANSFAC M01012. Thus SiteGA performed the best among models not derived from ChIP-Seq data, most probably because of its additional information relating distant sequence positions [12] (Figure 5A,B). To confirm that the selected EMSA score 0.25 is appropriate, we performed correlation coefficient calculations for two additional EMSA score values 0.17 and 0.34 (Additional file 2: Figure S2). One can see that for all models except the worst one (TRANSFAC M01261) CC computed for EMSA score 0.17 are notably lower than ones for EMSA scores 0.25 and 0.34. Among the latter two that provide similar CC values for nearly all models (Additional file 2: Figure S2) the first EMSA score (0.25) was chosen as less stringent.

We suspect that several factors are crucial in explaining performance of different models (Figure 5): (a) the training data-set, either ChIP-Seq derived or not; (b) a visible improvement for conventional PWMs if dinucleotide statistics are used along with extension of a matrix length (diChIPMunk vs. ChIPMunk, oPWM vs. Transfac PWM M01012); (c) accounting for possible distant dependencies (SiteGA vs. oPWM).

## Discussion

Computational methods of TFBS recognition provide important tools to analyze ChIP-Seq data [1, 46]. Many different tools were presented for motif discovery in ChIP-Seq data, including MEME-ChIP [47], RSAT peak-motifs [48], Dimont [49]. Yet, it is commonly PWMs from JASPAR and TRANSFAC that are applied for the approval of ChIP-Seq data. A few attempts were made to properly train complex models [50].

However, in the absence of additional experimental verification of TFBS models, it is difficult to estimate key model parameters, such as recognition thresholds, which would correctly separate true-positive from false-positive predictions. Ignorance of these thresholds, in turn, greatly complicates both the interpretation of ChIP-Seq data and the performance comparison of TFBS-prediction methods.

Application of several models for TFBS prediction in this study allowed us to resolve two important issues: (a) whether advanced pattern match models (oPWM, SiteGA), if properly trained on a limited curated TFBS set, can compete with pattern discovery models (ChIPMunk, diChIPMunk) trained on ChIP-Seq data; and (b) whether these advanced models can complement each other or commonly applied PWMs (four additional representatives from JASPAR and TRANSFAC were tested).

Using FoxA BSs as a case study, we have applied EMSA to determine thresholds for eight models of TFBS recognition. The use of these thresholds in the analysis of ChIP-Seq profiles for FoxA2 in adult mouse liver [24] and in human HepG2 cells [25] allowed us to reliably identify thousands of FoxA BSs within ChIP-Seq peaks and to conduct an adequate comparison of the computational models studied.

First of all, in some cases models for TFBS recognition are able to find trustworthy sites only in a small subset of peaks, e.g. 29% for TRANSFAC M01012 and 43% for oPWM (Figure 4A). This fraction is not what one should expect from a TRANSFAC model, and only performing the EMSA experiment allowed us to compute an accurate estimate. Application of such models for finding TFBSs in a major subset of ChIP-Seq peaks would require too low threshold value giving too many false positives. Hence, the threshold choice is the key point for getting trustworthy TFBS-recognition results from ChIP-Seq pipelines. It is important for different tasks, e.g. identification of reliable TFBSs in ChIP-Seq data or studying the ratio of direct and indirect protein binding to chromatin. While EMSA was used here for selecting thresholds, there could be other, less labor-intensive approaches, for example fluorescence correlation spectroscopy (FCS) [51]. It is noteworthy that once experimental verification is done for a particular protein, it could be re-used for defining the thresholds for any corresponding TFBS model and interpreting any ChIP-seq experiment for this protein, as we have demonstrated on the independent human FoxA2 ChIP-Seq dataset [25].

We conclude that models derived from ChIP-Seq usually outperformed the others. The important exception is SiteGA, most probably due to its more complex algorithm of information extraction and recognition. But this outperformance does not diminish the value of the models trained on independent data, which provide a way to verify the results obtained from a ChIP-Seq pipeline.

We have confirmed the supremacy of dinucleotide statistics for the TFBS models, showing its advantages for both classes of methods. An important component of this advantage comes from the informative data in the flanking regions of the TFBS “core” sequence. The consequence is typically longer matrix length, e.g. TRANSFAC M01012 (18 nt) vs. oPWM (32 nt) and ChIPMunk (20 nt) vs. diChIPMunk (28 nt).

The models based on different principles complement each other. Combined use of the models allowed better identification of FoxA sites in corresponding ChIP-Seq peaks. The best results for BS prediction in ChIP-Seq data were achieved by model combinations, e.g. SiteGA plus diChIPMunk, successfully identifying up to 90% of FoxA BS-containing loci in both the mouse and human ChIP-Seq data.

## Conclusion

Nowadays ChIP-Seq is the gold standard for studying TF-chromatin interactions *in vivo*. Detailed analysis of TFBS in ChIP-Seq data requires validated computational TFBS-recognition tools. The choice of the appropriate thresholds for TFBS models is one of the key steps that has been often underappreciated. We carried out an experimental study of FoxA binding to oligonucleotides, corresponding to predicted sites. This allowed us to determine the thresholds for several ChIP-Seq derived and conventional models, improving their ability to predict TFBSs. This also allowed us to compare models accurately using the independent control ChIP-Seq dataset, and conclude that (a) the weak ability to detect sites properly; (b) although pattern-discovery models derived from ChIP-Seq in general are better than conventionally derived pattern-match models, the latter valuably complement the former in annotation of ChIP-Seq data; (c) integration of different models allows detecting reliable sites in up to 90% of ChIP-Seq loci.

## Methods

### Datasets and site prediction tools

Two publicly available ChIP-Seq datasets for FoxA2 TF were used in this study: for adult mouse liver [24] (4455 mouse loci having base coverage no less than 15) and for human HepG2 cells (4367 human loci with base coverage no less than 10) [25]. The sequences were extracted from the mm8 and hg18 genome assemblies.

To train the pattern-matching models we gathered the set of 53 known BSs (Additional file 1: Table S2) of FoxA family TFs. The sequences were extracted from the TRRD database [52] and by manual literature mining. The selected BSs were confirmed by at least one of the following methods: (1) DNase I footprinting using purified protein, (2) electrophoretic mobility shift assay (EMSA) with purified protein, and (3) EMSA with nuclear extract and specific antibodies.

Sequences were aligned relative to the centrally located pattern TRTTTRYH (R = A/G, Y = C/T, H = A/C/G) [13] (Additional file 1: Table S2). The set of aligned sequences was then used as the training set for the optimized PWM (oPWM) and SiteGA models [12]. The construction of oPWM included the search for the optimal matrix length according to a resampling leave-one-out cross-validation test (Additional file 2: Figure S3). The SiteGA model takes into account statistical features of a binding site context reflecting putative structural interactions within the core and flanking regions of the site. It uses a genetic algorithm with a discriminant function of locally positioned dinucleotide frequencies. The performance test showed that the SiteGA model slightly outperformed oPWM (Additional file 2: Figure S4).

ChIPMunk [19] is a fast heuristic DNA motif discovery tool, which employs a greedy approach accompanied by bootstrapping. It is able to properly use the ChIP-Seq base coverage profile (the “peak shape”), producing high quality motifs as shown in several independent benchmark studies [21–23, 53]. ChIPMunk searches for the gapless multiple local alignment with the highest Kullback Discrete Information Content (KDIC) [19], under the common assumption of independence for neighboring nucleotides. An improved version of the algorithm (diChIPMunk [20]), uses a dinucleotide alphabet of 16 letters and the TFBS model accounting for the dependence between nucleotides in neighboring binding-site positions. A criterion for the alignment optimality, Kullback Dinucleotide Discrete Information Content (KDDIC), was constructed in the same way as KDIC using a dinucleotides alphabet. The matrix lengths for ChIPMunk and diChIPMunk were computed by the jack-knife optimization procedure similar to that for oPWM [12]. For ChIPMunk, diChIPMunk and oPWM matrix scores were rescaled as described for oPWM [12]. ChIPMunk trained on 4455 loci [24] resulted in 20 nt mono- and 28nt dinucleotide matrices (Additional file 2: Figure S5) which were in a good agreement with the TRTTTRYH pattern, determined from our training set as well as with the known FoxA binding consensus [54, 55].

### Plasmid construction and purification of GST fusion protein

The FKH DNA-binding domain of FoxA2 (nt 432–869) was amplified from rat genomic DNA by PCR using primers DBD-FoxA2_f (5′-GCGGAATTCCGCTCGGGACCCCAAGACGTA-3′, *Eco*RI site is underlined) and DBD-FoxA2_r (5′-GCGCTCGAGTCCCCGAGCTGAACCTGA-3′, *Xho*I site is underlined). The PCR product was then digested with *Xho*I and *Eco*RI, and cloned into the same sites of pGEX-4 T-1 vector (Pharmacia). The recombinant plasmid was transformed into *E. coli BL21 cells by electroporation*. Expression of GST-fused FKH domain of FoxA2 protein (GST-FKH-FoxA2) was induced by incubation with 0.1 mM isopropyl-β-D-thiogalactopyranoside for 3 h at 30°C. The GST-FKH-FoxA2 protein was purified using a glutathione-sepharose (Sigma) column according to the manufacturer’s protocol. The purity and size of the eluted protein were evaluated by separation on SDS-polyacrylamide gels and Coomassie Brilliant Blue staining.

### EMSA

The double-stranded synthetic oligonucleotide probes (containing predicted FoxA BSs) used for competition EMSAs are listed in Additional file 1: Table S1. A double-stranded TTR oligonucleotide containing a strong FoxA binding site from the transthyretin promoter [26] was labeled by filling-in the sticky ends with Klenow enzyme and [α-^32^P]dATP and used as DNA probe in EMSAs. In the competitive experiments, 2, 5 and 20 ng of unlabelled double-stranded oligonucleotide were added concurrently with 1 ng of ^32^P-labeled TTR probe to the reaction mixtures containing 25 mM HEPES (pH 7.6), 150 mM KCl, 0.2 mM EDTA, 0.2 mM EGTA, 10% glycerol, 1 mM dithiothreitol and 3 μg of GST-FKH-FoxA2 protein and allowed to incubate on ice for 15 min. Each gel run was supplemented with TTR self-competition as a positive control and a competition with PPAR oligonucleotide in order to control for non-specific binding. Immediately following the incubation, the bound complexes were separated from the free probe by electrophoresis on 4% nondenaturing polyacrylamide gels in 0.5× Tris-borate-EDTA buffer for 40 min at 4°C. After electrophoresis, the gels were dried and visualized by autoradiography on Retina medical x-ray film. Band intensities were detected and quantified using a Gel-Doc imaging system and Quantity One software (Bio-Rad). The respective concentration-response curves for each cold competitor were subjected to log-transformation and linear regression and resulting slope values were normalized to that of positive control (TTR self-competition) to achieve a rough estimate of the relative FoxA2-binding strength to the corresponding oligonucleotide (EMSA score, Additional file 2: Figure S1). For all models the threshold for “non-sites” was set at EMSA score 0.25 based on the observed technical error multiplied by 1.5.

## Electronic supplementary material

Additional file 1: **Hyperlinks to ChIP-Seq datasets** [24]**,**[25]**;** **Table S1.** Double-stranded synthetic oligonucleotide probes used in competition electrophoretic mobility shift assays (EMSAs) and their respective EMSA scores; **Table S2.** Training data for SiteGA and oPWM models, aligned FoxA BSs dataset, 53 BSs. (DOCX 24 KB)

Additional file 2: Figure S1: EMSA score distribution. X axis shows 64 oligonucleotides (potential FoxAsites) that were chosen for EMSA verification; oligonucleotides are shown in ascending order of EMSA scores. Y axis shows EMSA scores. **Figure S2.** Correlation coefficients (CC) for FoxA recognition models DiChIPMink, ChIPMunk, SiteGA, oPWM, MA0047.2 [41] and MA0148.1 [42] from JASPAR [8], M01261 [43] and M01012 [44] from TRANSFAC [7] FoxA recognition models. CC value were computed as described previously [45] for thresholds of respective recognition functions, selected to correspond EMSA score thresholds 0.17, 0.25 and 0.34. Higher CC value denotes better performance of a model. X axis lists recognition models; Y axis shows CC values. **Figure S3.** Recognition performance for dinucleotide PWMs as a function of a matrix width. The X and Y (logarithmic scale) axes respectively show the length of a matrix and a false-positive (FP) rate for a selected true-positive (TP) rate (shown in figure legend). **Figure S4.** The comparison of recognition performance between oPWM and SiteGA models. Both models are trained on the same set of 53 FoxA binding sites (Additional file 1: Table S2). True Positive (TP) and False Positive (FP) rates are fractions of training and background (shuffled) sets that were recognized at a selected threshold. The TP and FP rates were evaluated by a standard leave-one-out cross-validation test. **Figure S5.** Sequence LOGO representing TFBS models constructed by ChIPMunk (top) and diChIPMunk (bottom). Mononucleotide LOGO columns (top) are scaled according to a KDIC [19]. Dinucleotide motif LOGO of the diChIPMunk motif shows frequencies for dinucleotides (bottom, scaled according to a KDIDIC, [20]) formed by corresponding mononucleotide columns (top, scaled according to the KDIC). (ZIP 2 MB)
