# Supplementary material for: Application of experimentally verified transcription factor binding sites models for computational analysis of ChIP-Seq data
Source: BMC Genomics. 2014 Jan 29;15(1):80. doi: 10.1186/1471-2164-15-80 (PMC4234207; doi:10.1186/1471-2164-15-80)
Supplement: Supplementary file 1 — Additional file 1: Hyperlinks to ChIP-Seq datasets [24],[25]; Table S1. Double-stranded synthetic oligonucleotide probes used in competition electrophoretic mobility shift assays (EMSAs) and their respective EMSA scores; Table S2. Training data for SiteGA and oPWM models, aligned FoxA BSs dataset, 53 BSs. (DOCX 24 KB) [file 12864_2013_7008_MOESM1_ESM.docx]

Wederell’s dataset <http://www.bcgsc.ca/downloads/chiptf/mouse/FoxA2/FoxA2_ht10_mm8.wig.gz>, 11475 peaks [24].

Wallerman’s dataset

<http://nar.oxfordjournals.org/content/suppl/2009/10/12/gkp823.DC1/nar-01190-x-2009-File010.gz> 24080 peaks [25].

**s**

**Table S1.** Double-stranded synthetic oligonucleotide probes used in competition electrophoretic mobility shift assays (EMSAs) and their respective EMSA scores.

| Sub-sample name | Position of peak^1^ | Oligonucleotide sequence (forward strand)^2^ | EMSA score |
| --- | --- | --- | --- |
| Confirmed sites | chrX:6878436-6879017 | 5'-cagtTATCAATGTTTGTTTACCTTGTGGCT-3' | 1.65 |
|  | chr9:107745824-107746224 | 5'-cagtACTAGATGTTTACTTTAAGGGACCAG-3' | 1.30 |
|  | chr1:141375708-141377041 | 5'-cagtATTGGACATTGTTTATTTGTTTTGCC-3' | 1.21 |
|  | chr7:140707466-140708352 | 5'-cagtCCCTTCCCTTATTTACATTCCCGAGA-3' | 1.13 |
|  | chr9:122199103-122199591 | 5'-cagtGGATGCCTCTATTTGTTTAATCACAG-3' | 1.10 |
|  | chr11:85168610-85169132 | 5'-cagtGCGGCGCTGTGTTTACCTTCCGGGAG-3' | 1.08 |
|  | chr12:85340663-85341399 | 5'-cagtGGACCTTATTTACTTTCCTTAGCAAG-3' | 0.99 |
|  | chr9:107420590-107421345 | 5'-cagtATGTGTTGTTGTTGACTTTAAGGTCG-3' | 0.93 |
|  | chr15:54962925-54964176 | 5'-cagtTGGCCCCAGTATTTACATTTCTAACA-3' | 0.92 |
|  | chr4:3604382-3605358 | 5'-cagtGTGTTCCTATGTGTACACAGTACTTG-3' | 0.91 |
|  | chr19:12527263-12528126 | 5'-cagtCCTTGTGCTTATTTACATACCCCGCC-3' | 0.84 |
|  | chr1:7078701-7079739 | 5'-cagtAAGCCTCGCGTTGTTTGGTTAAGGGGCAGG-3' | 0.83 |
|  | chr10:75378448-75379244 | 5'-cagtAAGGTTATTATTATTTGTTTTATTTTGTTT-3' | 0.83 |
|  | chr6:34792983-34793652 | 5'-cagtGCAACAAGTTGTTTACACTCTTCATT-3' | 0.82 |
|  | chr4:61847631-61848007 | 5'-cagtGACAACCAATGTTGACTTAAAACCTT-3' | 0.82 |
|  | chr16:13816438-13817216 | 5'-cagtTGGAGTAGTTATTTACCTTAGCAGCC-3' | 0.81 |
|  | chr17:45901267-45902206 | 5'-cagtCCAGGGGACCTGTTTACCTTACATAG-3' | 0.70 |
|  | chr19:56926814-56927643 | 5'-cagtCACCGTTGTTTGTTCTGTGACAAATA-3' | 0.68 |
|  | chr3:83135377-83136301 | 5'-cagtTGCTGGCCTAATGTGGACACAGGGGGTTCC-3' | 0.64 |
|  | chr10:79387812-79388714 | 5'-cagtGTTGCCCGTCGTATTTGTATATGGTTTCCT-3' | 0.58 |
|  | chr6:121311851-121312840 | 5'-cagtCAGCTCCCATGTTTACCAGGCCTTGA-3' | 0.57 |
|  | chr7:100410756-100411345 | 5'-cagtTCACCTGAGGGTGTGTACACAGTCTCTCAG-3' | 0.56 |
|  | chrX:17319260-17320098 | 5'-cagtCGTGACCTTTGTTGACTCGCGTCAGT-3' | 0.53 |
|  | chr6:34792983-34793652 | 5'-cagtGTTCAGAGCTGTTTACTTGAACGCGA-3' | 0.53 |
|  | chr8:96902217-96902588 | 5'-cagtGGTCACCCTTTGGTTTACCTTTTAGGAACA-3' | 0.52 |
|  | chr12:8003233-8004042 | 5'-cagtCCCGGGCCTGTTTGCTTTTCCATACA-3' | 0.50 |
|  | chr7:44717527-44718191 | 5'-cagtCTCCTCCATTGTGTACACTCCCCCTC-3' | 0.49 |
|  | chr11:45798706-45799259 | 5'-cagtAGGTTTCTCTATTGACTCTATTGCCT-3' | 0.49 |
|  | chr19:38189585-38190904 | 5'-cagtTTGGGTCAATATTTATTCTTTCATTT-3' | 0.48 |
|  | chr17:32151628-32152174 | 5'-cagtGGGACCTGTCTTGTTGACCCATCACGTCAC-3' | 0.47 |
|  | chr19:44039737-44040443 | 5'-cagtCTTGAGGCCTGTTTACCGTCCAGTTA-3' | 0.43 |
|  | chr7:29476412-29477261 | 5'-cagtACTCATCCTGCTATTGACAGTCGGATATAT-3' | 0.40 |
|  | chr12:77880754-77881604 | 5'-cagtCCAGACTCGCTTGTTGACAGAGCTCAGGGC-3' | 0.38 |
|  | chr3:116606401-116607138 | 5'-cagtATTTATTTGTATGTATTTTGAGACAG-3' | 0.38 |
|  | chr15:82621996-82622387 | 5'-cagtGGAACCTCTGATGTTGACTTTCCCTTGATT-3' | 0.38 |
|  | chr1:159362347-159362799 | 5'-cagtTGGGCATATTTGCTCAGAACCTGGTA-3' | 0.37 |
|  | chr8:112454327-112455374 | 5'-cagtGAGGCGGGGTGTTGACTCTTACGCCA-3' | 0.33 |
|  | chr15:82621996-82622387 | 5'-cagtCATATAGGCTCTGTTTGAACATTAATTAAC-3' | 0.31 |
|  | chr3:94456673-94457497 | 5'-cagtCTGCCCTGCTGTTTACCAGCTGGAGA-3' | 0.29 |
|  | chr15:59478046-59478654 | 5'-cagtGTGAGTGTGTGTGTGTATACAGCGTGAGTC-3' | 0.26 |
|  | chr7:15773405-15773876 | 5'-cagtCTGCGGCGCCAGATTTACACAGCGCGAGGA-3' | 0.25 |
| Non-confirmed sites | chr7:83767529-83768177 | 5'-cagtAGGGTCAAGGTAGTTTGCATATCTTCCAAC-3' | 0.24 |
|  | chr1:154160175-154161537 | 5'-cagtTCATGCCACAGTTTACTCTGGTCCCT-3' | 0.24 |
|  | chr7:119551431-119552250 | 5'-cagtAAACTCTGCTGTGTGTACAGATAACATTAC-3' | 0.22 |
|  | chr5:30822343-30823153 | 5'-cagtTGCCTTTCTGTTTGTTGTTTTGGAGG-3' | 0.21 |
|  | chr17:25500984-25501953 | 5'-cagtTTCAAAGCTTGTTGACGGAGAGAAAT-3' | 0.20 |
|  | chr5:139643316-139643686 | 5'-cagtCCCTATCTGTGTTTACAGCTGGTTTC-3' | 0.18 |
|  | chr9:102865800-102866163 | 5'-cagtAGCGCCTGCAGTTTACCTTGGCTCCG-3' | 0.13 |
|  | chr8:87127281-87127682 | 5'-cagtCTCCCGGCCGGGGTTGACTCTCTCTGAGCT-3' | 0.11 |
|  | chr7:59095749-59096651 | 5'-cagtAGCCCCTCCTGTGTTGTCTTCCAGTAGATG-3' | 0.09 |
|  | chr8:13025407-13026283 | 5'-cagtCTGAGAAACCTTGTTGATCTCAGGCAACCC-3' | 0.07 |
|  | chr12:85340663-85341399 | 5'-cagtCGTCTTCCCTGTGTGCTTACTATGCA-3' | 0.07 |
|  | chr3:94456673-94457497 | 5'-cagtCCTGGCTTTCCTGCTTACCCAACAACCGCT-3' | 0.07 |
|  | chr16:22806861-22807941 | 5'-cagtCTCTGCCCCTCTATTGGTCTAGCTCTCCAA-3' | 0.06 |
|  | chr12:77880754-77881604 | 5'-cagtTGCGCCCCGGGTGTTGGCAGCGAGAAGTGG-3' | 0.03 |
|  | chr15:91019750-91020736 | 5'-cagtCTCTGAGTGACTGTTTTCTGTAATACCCAA-3' | 0.01 |
|  | chr17:74394072-74394898 | 5'-cagtCCGGGATTGGCTGTTTGTAATCTGCTCGAC-3' | 0.00 |
|  | chr7:140707466-140708352 | 5'-cagtGGCATCCGACTGATTTACTGAGCTGGTTCC-3' | 0.00 |
|  | chr6:5446118-5446863 | 5'-cagtGCGTGCATTGGTGTTCGCTCAGAGCGGGAC-3' | 0.00 |
|  | chr8:96902217-96902588 | 5'-cagtTATCTGTACTTTATTTTCAAACGCTATAAT-3' | -0.01 |
|  | chr13:40870570-40871664 | 5'-cagtCAGTGGAAGGCTGTTAACTGTGCCTGCTGC-3' | -0.02 |
|  | chr8:13025407-13026283 | 5'-cagtGCTAGATCCAGTTTTTACACAGAAAGTTTC-3' | -0.06 |
|  | chr7:44970992-44972284 | 5'-cagtACCCCAGGATTTGTGGGCTGTGGGTGTGGC-3' | -0.11 |
|  | chr7:26648074-26648575 | 5'-cagtAGAGGTCTTCCTCTTTGCCCATGTTGTGGG-3' | -0.16 |
| P.C. | TTR | 5'-cagtCGAGTTGACTAAGTCAATAATCAGAATCAGTCG-3' | 1.00 |
| N.C. | PPAR | 5'-сgatCAAAACTAGGTCAAAGGTCA-3' | 0.00 |

^1^ position of peak containing potential site in mm8 mouse genome release according to [24];

^2^ 5'-overhanging nucleotides introduced for labeling are shown in lowercase. P.C. – positive control site; N.C. – negative control site.

**Table S2.** Training data for SiteGA and oPWM models, aligned FoxA BSs dataset, 53 BSs.

| # | Gene | Species | Site position respective to annotated start of transcription^1^ | Training dataset of sequences, capital letters denote locations of consensus TRTTTRYH |
| --- | --- | --- | --- | --- |
| 1 | SQRDL | Rat | –193..-184 | cggaaacttttTATTTACTcttatacctgttt |
| 2 | PROC | Human | –33..-22 | ataaccacaaaTATTTGCTtggccctcagcac |
| 3 | ALDOB | Rat | -126..-104 | cagtagggaggTGTTTATTcaataactctgat |
| 4 | TAT | Rat | -5399..-5364 | tagcaccacctTATTTGTTttgtgttttgcat |
| 5 | TAT | Rat | -5377..-5345 | taaggtggtgcTATTTGCTaaactccgtgagt |
| 6 | TAT | Rat | -5322..-5291 | aaatcttcatcTGTTTGCAgactgaggtgcct |
| 7 | TAT | Rat | -2487..-2465 | tctagctacttTATTTGCAatagaaaatctga |
| 8 | TAT | Rat | -2440..-2414 | tacgcaggactTGTTTGTTctagtcttgttgg |
| 9 | TAT | Rat | -10514..-10484 | acgtttctcaaTATTTGCTctggcagagggag |
| 10 | PFKFB1 | Rat | -132..-112 | ctttggtctttTATTTGCAtactctactagtc |
| 11 | CDX2 | Mouse | 4716..4728 | aaagggtttttTGTTTGTTtgttttttgtttt |
| 12 | CDH1 | Mouse | -551..-563 | ggtagctttttTGTTTGTTtgttttccaactc |
| 13 | NKX2-1 | Human | -135..-124 | aaacttaaaggTGTTTACCttgtcatcagcat |
| 14 | POL | Virus^2^ | -40..-26 | atctcctccatTGTTTGCAcatggtctaatac |
| 15 | G6PC | Human | -139..-133 | caccctgaacaTGTTTGCAtcaacctactggt |
| 16 | AMBP | Human | -2753..-2733 | tttactctctcTGTTTGCTctggttaataatc |
| 17 | ALPI | Human | -953..-941 | tgttcgtgcgaTGTTTGTTcttttgcactggt |
| 18 | FABP2 | Rat | -383..-371 | ttcattcttgtTGTTTGTCttttggctggagt |
| 19 | APOB | Human | 863..873 | cgggcctgaccTGTTTGCTtttctacactggc |
| 20 | APOB | Human | 894..904 | catcactgtccTGTTTATCagtgactagtcat |
| 21 | CPS1 | Rat | 335..357 | aaaatcatcagTGTTTGCTcttgagttgaaaa |
| 22 | SFTBP | Human | -92..-72 | agcgacctcagTGTTTGTCtttgctctgaaga |
| 23 | FOXA2 | Mouse | -93..-85 | acctactgcccTGTTTGTTttagttacgaaat |
| 24 | MSH2 | Human | –986..-974 | gtttgtcttttTATTTATTttattttgtattt |
| 25 | VTN | Mouse | -41.. -11 | tttctgcccttTATTTGCTcatcctctggccc |
| 26 | KLK3 | Human | -4028..-4005 | tgtttcaaggaTGTTTGTAaagcaggcatcct |
| 27 | ALAS1 | Rat | -419..-380 | ttgcttggtttTGTTTGCAtcgatgacaggga |
| 28 | SLC10A1 | Human | -76..-34 | cataacccttcTATTTGCCcagagcttttgtc |
| 29 | FGB | Human | -165..-140 | ccagcaaagctTATTTACTtgtcatacaacta |
| 30 | ONECUT1 | Mouse | 4903..4926 | aacctgggccgTGTTTGCTgtgagtggccttg |
| 31 | GCG | Mouse | -97..-77 | caaaaccccatTATTTACAgatgagaaattta |
| 32 | CEBPA | Human | 26301..26321 | tccagcaggccTGTTTACTcagaggcccacga |
| 33 | G6PC2 | Mouse | -246..-241 | cagttggatcgTGTTTGCTtgcaatttttcat |
| 34 | MAFA | Mouse | -7943..-7910 | gagctgtaaaaTGTTTACAgaaagggtcgttt |
| 35 | SCGB1A1 | Rabbit | -136..-120 | agaaaagggaaTATTTACTtatcccaccaagt |
| 36 | SCGB1A1 | Rabbit | -105..-86 | cttgactgcatTATTTACTtgggcattgactt |
| 37 | PC | Rat | -817..-776 | gaggacttattTGTTTACTgtctggtctccta |
| 38 | F2 | Human | -895..-871 | acttagactaaTATTTGCCttgggtactgcaa |
| 39 | COL18A1 | Mouse | -2794..-2775 | tggtatccagcTGTTTGCAgctggactctgag |
| 40 | PROS1 | Human | -282..-258 | tgtctcagcagTGTTTACTaggcctccaacac |
| 41 | KCNJ11 | Mouse | -1310..-1266 | gatcctacatcTATTTATTtacttatttgttt |
| 42 | LPL | Mouse | -48..-29 | gtgatgagtctTATTTGCAtatttccagtcac |
| 43 | UCP2 | Mouse | 2780..2799 | gccaggttgccTGTTTGTTttcctccagtgtc |
| 44 | SLC25A1 | Human | -1104..-1081 | cacctggacaaTATTTATTtttgctgaaaccc |
| 45 | SLCO1B3 | Human | -38..-24 | agttggcttttTATTTGTTtgtttttatgagt |
| 46 | SFTPD | Human | -288..-256 | atcagtgtctgTATTTATAgatgtctagaaat |
| 47 | UGT2B15 | Human | -217..-208 | gtgttcttttaTGTTTACAattactctagtca |
| 48 | AHSG | Mouse | -134/-115 | atgtcctaactTATTTGCTtttccagagctgc |
| 49 | POL | Virus^3^ | 1124..1139 | ggttcatgtacTGTTTACTtagaaaggccttg |
| 50 | CYP3A4 | Human | -1738..-1710 | ccaagcatgttTATTTGTCtcctgcttcacta |
| 51 | UCP2 | Mouse | -1760..-1749 | taaaaaagattTATTTATTttatgtatatgag |
| 52 | PDX1 | Mouse | -6264..-6233 | gggctctggctTATTTACTttctgttctccca |
| 53 | TBX1 | Mouse | -13435..-13423 | gaggagcagccTGTTTGTTttgccagatctgt |

^1^ Location respective to transcription start sites from RefSeq database

^2^ Duck hepatitis B virus, AC M60677;
^3^ Human hepatitis B virus isolate P41, AC GQ477480.
