# Supplementary figures and images for: Application of experimentally verified transcription factor binding sites models for computational analysis of ChIP-Seq data
Source: BMC Genomics. 2014 Jan 29;15(1):80. doi: 10.1186/1471-2164-15-80 (PMC4234207; doi:10.1186/1471-2164-15-80)

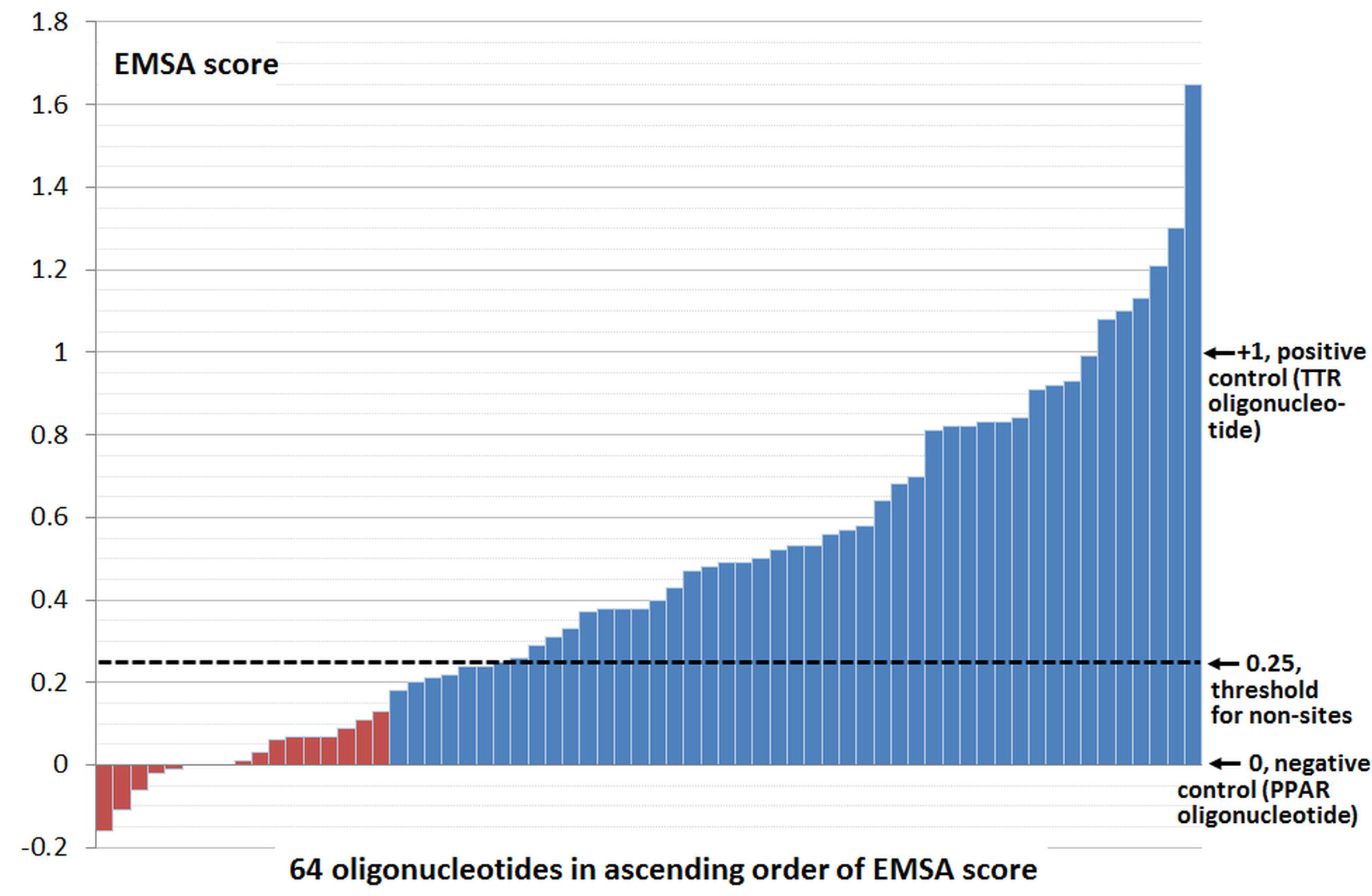

Supplement: Supplementary file 2 — Additional file 2: Figure S1: EMSA score distribution. X axis shows 64 oligonucleotides (potential FoxAsites) that were chosen for EMSA verification; oligonucleotides are shown in ascending order of EMSA scores. Y axis shows EMSA scores. Figure S2. Correlation coefficients (CC) for FoxA recognition models DiChIPMink, ChIPMunk, SiteGA, oPWM, MA0047.2 [41] and MA0148.1 [42] from JASPAR [8], M01261 [43] and M01012 [44] from TRANSFAC [7] FoxA recognition models. CC value were computed as described previously [45] for thresholds of respective recognition functions, selected to correspond EMSA score thresholds 0.17, 0.25 and 0.34. Higher CC value denotes better performance of a model. X axis lists recognition models; Y axis shows CC values. Figure S3. Recognition performance for dinucleotide PWMs as a function of a matrix width. The X and Y (logarithmic scale) axes respectively show the length of a matrix and a false-positive (FP) rate for a selected true-positive (TP) rate (shown in figure legend). Figure S4. The comparison of recognition performance between oPWM and SiteGA models. Both models are trained on the same set of 53 FoxA binding sites (Additional file 1: Table S2). True Positive (TP) and False Positive (FP) rates are fractions of training and background (shuffled) sets that were recognized at a selected threshold. The TP and FP rates were evaluated by a standard leave-one-out cross-validation test. Figure S5. Sequence LOGO representing TFBS models constructed by ChIPMunk (top) and diChIPMunk (bottom). Mononucleotide LOGO columns (top) are scaled according to a KDIC [19]. Dinucleotide motif LOGO of the diChIPMunk motif shows frequencies for dinucleotides (bottom, scaled according to a KDIDIC, [20]) formed by corresponding mononucleotide columns (top, scaled according to the KDIC). (ZIP 2 MB) [file 12864_2013_7008_MOESM2_ESM.zip › 1322440626101266_add2.png]

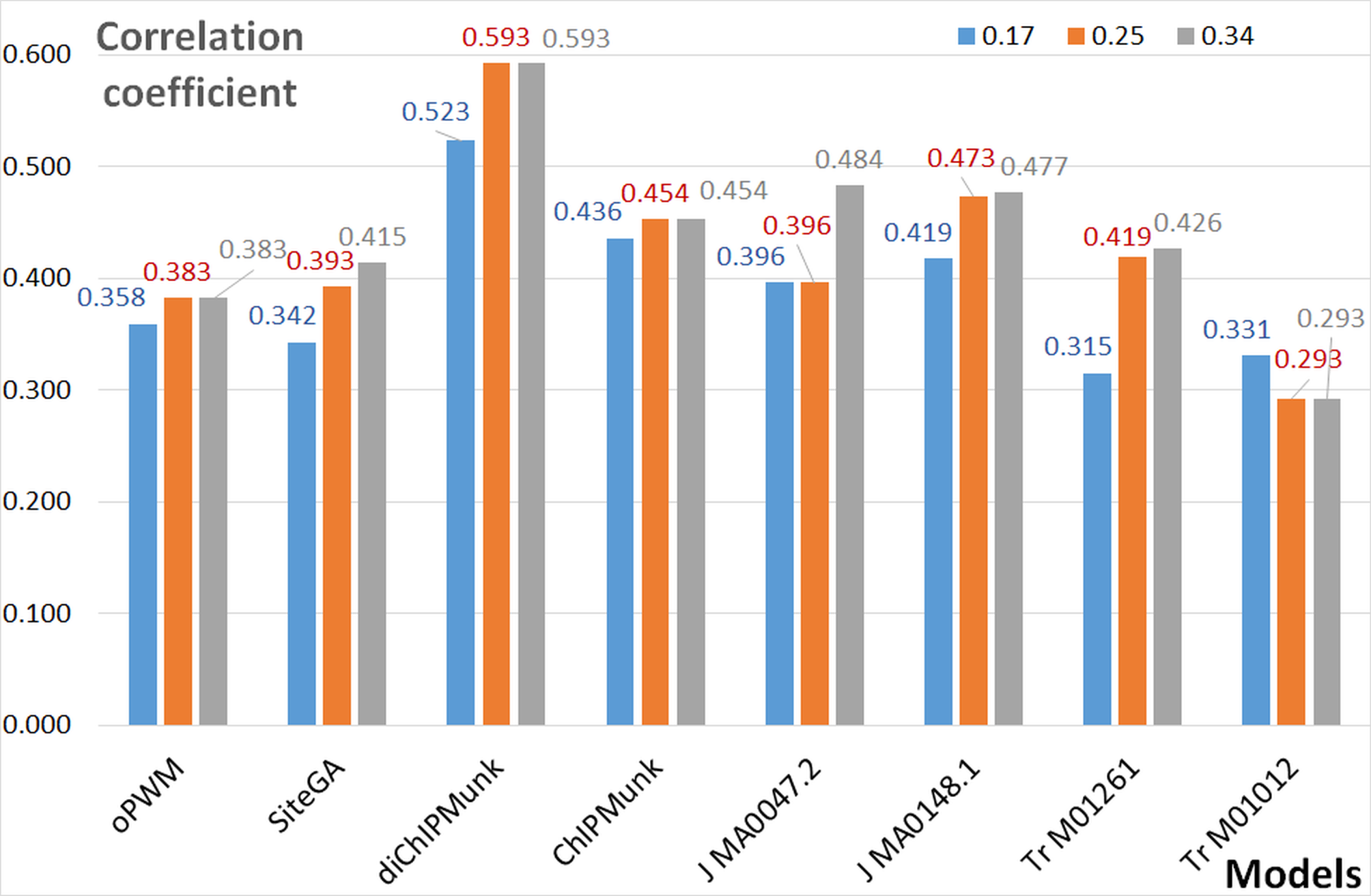

Supplement: Supplementary file 2 — Additional file 2: Figure S1: EMSA score distribution. X axis shows 64 oligonucleotides (potential FoxAsites) that were chosen for EMSA verification; oligonucleotides are shown in ascending order of EMSA scores. Y axis shows EMSA scores. Figure S2. Correlation coefficients (CC) for FoxA recognition models DiChIPMink, ChIPMunk, SiteGA, oPWM, MA0047.2 [41] and MA0148.1 [42] from JASPAR [8], M01261 [43] and M01012 [44] from TRANSFAC [7] FoxA recognition models. CC value were computed as described previously [45] for thresholds of respective recognition functions, selected to correspond EMSA score thresholds 0.17, 0.25 and 0.34. Higher CC value denotes better performance of a model. X axis lists recognition models; Y axis shows CC values. Figure S3. Recognition performance for dinucleotide PWMs as a function of a matrix width. The X and Y (logarithmic scale) axes respectively show the length of a matrix and a false-positive (FP) rate for a selected true-positive (TP) rate (shown in figure legend). Figure S4. The comparison of recognition performance between oPWM and SiteGA models. Both models are trained on the same set of 53 FoxA binding sites (Additional file 1: Table S2). True Positive (TP) and False Positive (FP) rates are fractions of training and background (shuffled) sets that were recognized at a selected threshold. The TP and FP rates were evaluated by a standard leave-one-out cross-validation test. Figure S5. Sequence LOGO representing TFBS models constructed by ChIPMunk (top) and diChIPMunk (bottom). Mononucleotide LOGO columns (top) are scaled according to a KDIC [19]. Dinucleotide motif LOGO of the diChIPMunk motif shows frequencies for dinucleotides (bottom, scaled according to a KDIDIC, [20]) formed by corresponding mononucleotide columns (top, scaled according to the KDIC). (ZIP 2 MB) [file 12864_2013_7008_MOESM2_ESM.zip › 1322440626101266_add3.png]

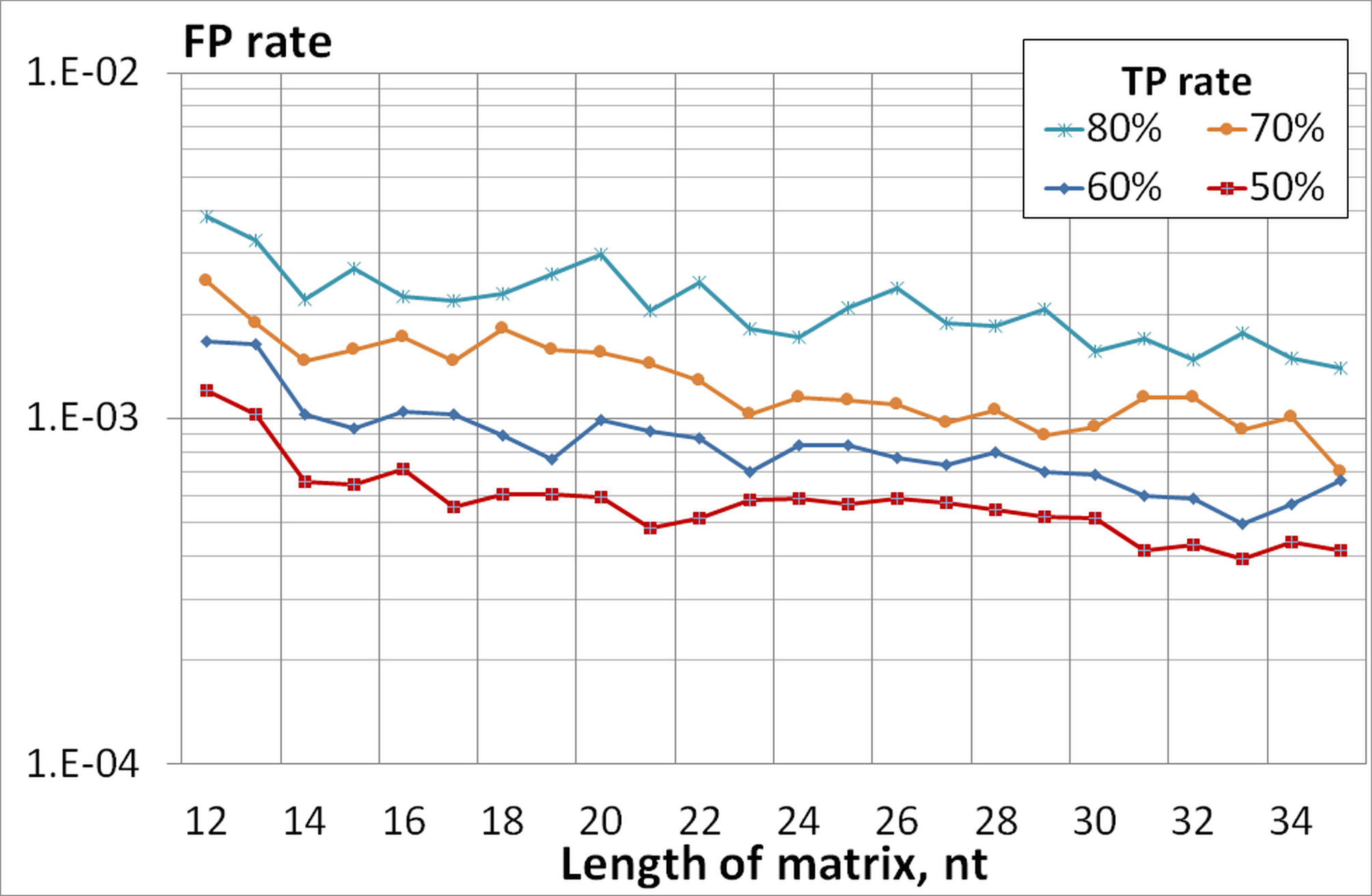

Supplement: Supplementary file 2 — Additional file 2: Figure S1: EMSA score distribution. X axis shows 64 oligonucleotides (potential FoxAsites) that were chosen for EMSA verification; oligonucleotides are shown in ascending order of EMSA scores. Y axis shows EMSA scores. Figure S2. Correlation coefficients (CC) for FoxA recognition models DiChIPMink, ChIPMunk, SiteGA, oPWM, MA0047.2 [41] and MA0148.1 [42] from JASPAR [8], M01261 [43] and M01012 [44] from TRANSFAC [7] FoxA recognition models. CC value were computed as described previously [45] for thresholds of respective recognition functions, selected to correspond EMSA score thresholds 0.17, 0.25 and 0.34. Higher CC value denotes better performance of a model. X axis lists recognition models; Y axis shows CC values. Figure S3. Recognition performance for dinucleotide PWMs as a function of a matrix width. The X and Y (logarithmic scale) axes respectively show the length of a matrix and a false-positive (FP) rate for a selected true-positive (TP) rate (shown in figure legend). Figure S4. The comparison of recognition performance between oPWM and SiteGA models. Both models are trained on the same set of 53 FoxA binding sites (Additional file 1: Table S2). True Positive (TP) and False Positive (FP) rates are fractions of training and background (shuffled) sets that were recognized at a selected threshold. The TP and FP rates were evaluated by a standard leave-one-out cross-validation test. Figure S5. Sequence LOGO representing TFBS models constructed by ChIPMunk (top) and diChIPMunk (bottom). Mononucleotide LOGO columns (top) are scaled according to a KDIC [19]. Dinucleotide motif LOGO of the diChIPMunk motif shows frequencies for dinucleotides (bottom, scaled according to a KDIDIC, [20]) formed by corresponding mononucleotide columns (top, scaled according to the KDIC). (ZIP 2 MB) [file 12864_2013_7008_MOESM2_ESM.zip › 1322440626101266_add4.png]

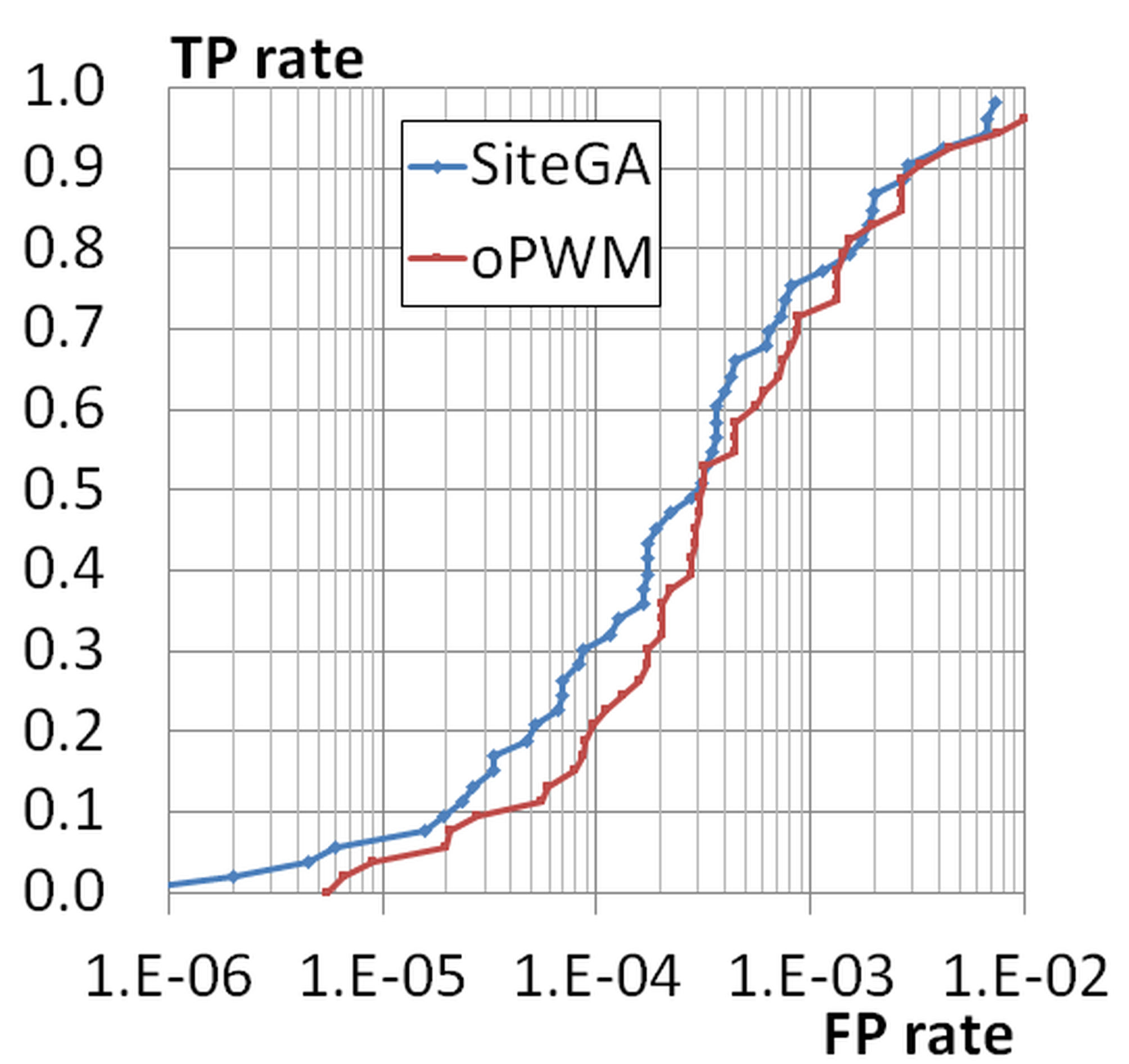

Supplement: Supplementary file 2 — Additional file 2: Figure S1: EMSA score distribution. X axis shows 64 oligonucleotides (potential FoxAsites) that were chosen for EMSA verification; oligonucleotides are shown in ascending order of EMSA scores. Y axis shows EMSA scores. Figure S2. Correlation coefficients (CC) for FoxA recognition models DiChIPMink, ChIPMunk, SiteGA, oPWM, MA0047.2 [41] and MA0148.1 [42] from JASPAR [8], M01261 [43] and M01012 [44] from TRANSFAC [7] FoxA recognition models. CC value were computed as described previously [45] for thresholds of respective recognition functions, selected to correspond EMSA score thresholds 0.17, 0.25 and 0.34. Higher CC value denotes better performance of a model. X axis lists recognition models; Y axis shows CC values. Figure S3. Recognition performance for dinucleotide PWMs as a function of a matrix width. The X and Y (logarithmic scale) axes respectively show the length of a matrix and a false-positive (FP) rate for a selected true-positive (TP) rate (shown in figure legend). Figure S4. The comparison of recognition performance between oPWM and SiteGA models. Both models are trained on the same set of 53 FoxA binding sites (Additional file 1: Table S2). True Positive (TP) and False Positive (FP) rates are fractions of training and background (shuffled) sets that were recognized at a selected threshold. The TP and FP rates were evaluated by a standard leave-one-out cross-validation test. Figure S5. Sequence LOGO representing TFBS models constructed by ChIPMunk (top) and diChIPMunk (bottom). Mononucleotide LOGO columns (top) are scaled according to a KDIC [19]. Dinucleotide motif LOGO of the diChIPMunk motif shows frequencies for dinucleotides (bottom, scaled according to a KDIDIC, [20]) formed by corresponding mononucleotide columns (top, scaled according to the KDIC). (ZIP 2 MB) [file 12864_2013_7008_MOESM2_ESM.zip › 1322440626101266_add5.png]

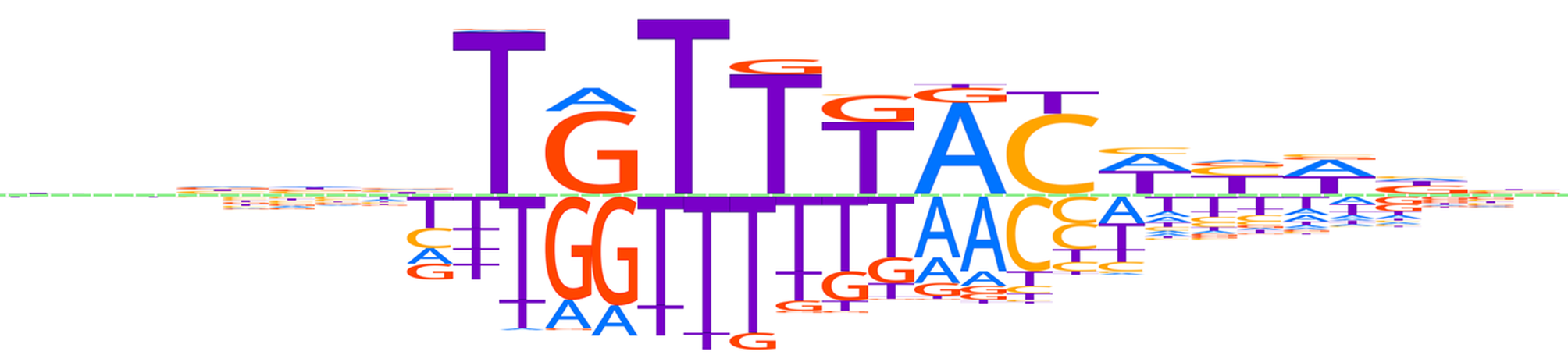

Supplement: Supplementary file 2 — Additional file 2: Figure S1: EMSA score distribution. X axis shows 64 oligonucleotides (potential FoxAsites) that were chosen for EMSA verification; oligonucleotides are shown in ascending order of EMSA scores. Y axis shows EMSA scores. Figure S2. Correlation coefficients (CC) for FoxA recognition models DiChIPMink, ChIPMunk, SiteGA, oPWM, MA0047.2 [41] and MA0148.1 [42] from JASPAR [8], M01261 [43] and M01012 [44] from TRANSFAC [7] FoxA recognition models. CC value were computed as described previously [45] for thresholds of respective recognition functions, selected to correspond EMSA score thresholds 0.17, 0.25 and 0.34. Higher CC value denotes better performance of a model. X axis lists recognition models; Y axis shows CC values. Figure S3. Recognition performance for dinucleotide PWMs as a function of a matrix width. The X and Y (logarithmic scale) axes respectively show the length of a matrix and a false-positive (FP) rate for a selected true-positive (TP) rate (shown in figure legend). Figure S4. The comparison of recognition performance between oPWM and SiteGA models. Both models are trained on the same set of 53 FoxA binding sites (Additional file 1: Table S2). True Positive (TP) and False Positive (FP) rates are fractions of training and background (shuffled) sets that were recognized at a selected threshold. The TP and FP rates were evaluated by a standard leave-one-out cross-validation test. Figure S5. Sequence LOGO representing TFBS models constructed by ChIPMunk (top) and diChIPMunk (bottom). Mononucleotide LOGO columns (top) are scaled according to a KDIC [19]. Dinucleotide motif LOGO of the diChIPMunk motif shows frequencies for dinucleotides (bottom, scaled according to a KDIDIC, [20]) formed by corresponding mononucleotide columns (top, scaled according to the KDIC). (ZIP 2 MB) [file 12864_2013_7008_MOESM2_ESM.zip › 1322440626101266_add6.png]
